# Health Outcomes Associated with Blood Lipid Levels and Korean Medicine Utilization in Elderly Population from the NHIS Database: A Retrospective Cohort Study

**DOI:** 10.3390/jcm15083150

**Published:** 2026-04-20

**Authors:** Seungcheol Hong, Ji-cheon Jeong, Dong-jun Choi

**Affiliations:** 1 Phil Hospital of Korean Medicine Cheongju, Cheongju 28377, Republic of Korea; chol1992@naver.com; 2 Department of Internal Korean Medicine, College of Korean Medicine, Dongguk University, Goyang 10326, Republic of Korea; 3 Department of Internal Korean Medicine, Dongguk University Ilsan Oriental Hospital, Dongguk University Medical Centre, Goyang 10326, Republic of Korea

**Keywords:** elderly, blood lipid level, Korean medicine, health outcome, retrospective cohort study

## Abstract

**Background**: The elderly are vulnerable to chronic diseases and altered lipid metabolism, leading to poor outcomes, including mortality. We investigated the association between Korean Medicine (KM) utilization, blood lipid levels, and health outcomes using the National Health Insurance Service Sample Cohort (NHIS-NSC) database. **Methods**: This retrospective cohort study included elderly participants who underwent health examinations (2009–2010). Participants were divided into KM and non-KM groups and matched 1:1 using propensity score matching (PSM) for age, sex, income, and comorbidities. Primary outcomes were mortality and disease diagnosis; secondary outcomes included medical spending and utilization. **Results**: After PSM, 13,044 subjects were analyzed. KM utilization was associated with a significantly lower risk of all-cause mortality (HR 0.93; 95% CI 0.87–1.00; *p* = 0.048). However, the hypolipidemia subgroup showed no significant differences in all-cause mortality and medical expenses compared to other lipid status subgroups. While the KM group showed a higher incidence of disease diagnosis (HR 1.09; 95% CI 1.04–1.14; *p* < 0.001), this may reflect increased healthcare engagement and proactive health-seeking behavior. Subgroup analysis revealed that statin users in the KM group had a significantly reduced mortality risk (HR 0.91; 95% CI 0.84–0.99; *p* = 0.022). Medical expenses and utilization were higher in the KM group. Being underweight or aged over 85 was associated with higher mortality. **Conclusions**: KM utilization is associated with reduced all-cause mortality after propensity score matching, particularly among statin users. Although KM users had a higher cumulative incidence of disease diagnosis, this potentially reflects increased diagnostic opportunities from prolonged survival. Hypolipidemia, underweight, and late-elderly status remain significant risk factors associated with frailty. KM may support improved survival in the elderly, warranting further prospective studies.

## 1. Introduction

As of July 2024, the population of individuals aged 65 years and older in the Republic of Korea is projected to exceed 10 million, marking the country’s entry into a super-aged society, where the elderly population constitutes over 20% of the total population [1]. According to a survey conducted by the Ministry of Health and Welfare, 84% of older adults suffer from chronic diseases such as cerebrovascular diseases, heart disease, cancer, respiratory diseases, and musculoskeletal disorders, with 54.9% having two or more comorbidities. These chronic conditions not only contribute to a high prevalence of illness but are also identified as leading causes of mortality in this demographic [2,3].

Given the anticipated increase in the elderly population, there is a pressing need for research on the factors that influence their health outcomes. Dyslipidemia, which encompasses conditions such as hypercholesterolemia, hypertriglyceridemia, and hyper-low-density lipoprotein (LDL) cholesterolemia, significantly affects various health outcomes. In 2020, the prevalence of hypercholesterolemia in the Republic of Korea was 24%, and dyslipidemia affected 40.2% of the population [4,5]. A cohort study conducted by the American Heart Association and the American College of Cardiology (AHA/ACC) observed that the ten-year incidence of ischemic heart disease was influenced by age, blood pressure, blood lipid levels, smoking, and diabetes, with elevated lipid levels being a key risk factor [6]. Additionally, hyperlipidemia in younger populations increases the long-term risk of heart disease.

In contrast, hypolipidemia refers to low total cholesterol (TC) and LDL cholesterol (LDL-C) levels, being within the 5th percentile of a normal population sample. Hypolipidemia can be congenital, as in abetalipoproteinemia and familial hypobetalipoproteinemia, or acquired, often due to the lipid-lowering medications used for hyperlipidemia. Data from the National Health and Nutrition Examination Survey (NHANES) indicated that blood lipid levels tended to increase from vicenarians to sexagenarians, followed by a decline in septuagenarians [7]. Although the underlying mechanisms are not fully understood, hypolipidemia may lead to deficiencies in thyroid, adrenal, and sex hormones. Moreover, lipid-lowering medications have been linked to frailty in elderly individuals [8]. Therefore, it is essential to investigate the relationship between blood lipid levels, including hypolipidemia, and health outcomes in older adults.

From the perspective of Korean Medicine, the elderly are particularly vulnerable to frailty, which is associated with *consumptive disease*, *kidney deficiency*, *Yin deficiency*, and *Yang deficiency* [9,10]. Korean Medicine treatments focus on nourishing the body, replenishing fluids, and warming the body to manage and prevent age-related diseases [11,12]. This study aimed to examine the impact of Korean Medicine on the health outcomes in the elderly population, using real-world data from the National Health Insurance Service (NHIS) data on Korean Medicine clinic visits.

We hypothesized that health outcomes would vary significantly based on blood lipid levels and the utilization of Korean Medicine among the elderly. Thus, this research aimed to conduct a retrospective cohort study utilizing secondary health care data from the NHIS, analyzing a sample cohort representing approximately 5% of the insured population. This study assessed the relationship between blood lipid levels and health outcomes in elderly individuals, with a focus on the presence or absence of dyslipidemia through a 3-arm cohort comparison. Health outcomes evaluated include disease morbidity (e.g., stroke and ischemic heart disease), mortality rates, survival time, and socioeconomic impacts such as medical costs.

## 2. Methods

### 2.1. Data Source

This study analyzed data from the NHIS National Sample Cohort (NHIS-NSC) Database. As of January 2025, the NHIS provides coverage for the entire population of 51.68 million people in the Republic of Korea. The database contains tables of collected information, including general insurance information, birth and death records, medical service logs, expenses, diagnoses, medications, and health examinations. The NHIS-NSC Database established a million random, stratified, pseudonymized samples extracted from the population data in 2006 and compiled longitudinal cohort data between 2002 and 2019. It represents approximately 2.2% of the entire Korean population [13].

### 2.2. Study Design and Definitions

This retrospective cohort study compared the effects of Korean Medicine utilization on elderly subjects (aged 65 or older) who underwent health examinations between 2009 and 2010. The cohort was stratified by age, blood lipid levels, and statin use, and then divided into two groups based on Korean Medicine utilization.

#### 2.2.1. Inclusion Criteria

Participants were individuals aged 65 years or older who were enrolled in the NHIS and were extracted from the NHIS-NSC 2.0 database. They must have had health examination data available from the health examination table (40T), including blood lipid panels of TC, HDL cholesterol (HDL-C), LDL-C, and triglycerides (TG) from 2009 to 2010. As NHIS provides biennial health examinations, data from both odd- and even-numbered years were included.

**Figure 1:**
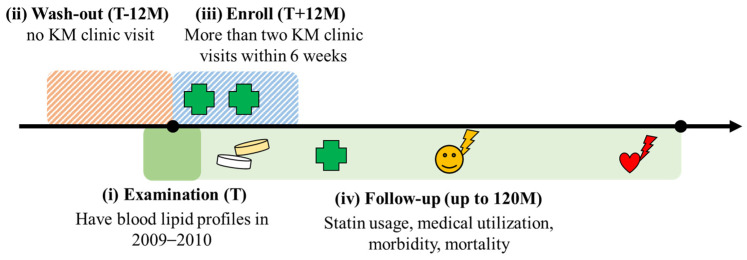
Operational definition for inclusion criteria. (**i**) Examination (T): Subjects who had blood lipid profiles in the health examination data from 2009 to 2010 were screened. (**ii**) Wash-out (T-12M): Subjects who had any Korean Medicine clinic visits within 12 months prior to inclusion were excluded for wash-out. (**iii**) Enroll (T+12M): Subjects who had more than two sessions of Korean Medicine clinic visits within a 6-week period occurring within the first 12 months after inclusion were enrolled for analysis. (**iv**) Follow-up (up to 120M): Surveillance of statin usage, medical utilization, morbidity, and mortality up to 120 months from the baseline was performed and analyzed.

A 12-month wash-out period was applied to identify Korean Medicine users (KM user), and the enrollment period for Korean Medicine utilization was set to 12 months after the health examination. Based on discussions between clinical experts (D. Choi and J Jeong) and researchers (S. Hong), and with reference to studies that conducted similar designs and objectives, patients who had records of visiting a Korean Medicine clinic for at least two sessions within 6 weeks during the enrollment period were assigned to the KM user group, as indicated in the medical treatment history table (20T). Patients who had no records of visiting a Korean Medicine clinic were assigned to the non-KM user group for the control. The types and frequencies of the Korean medical treatments were not restricted (Figure 1).

#### 2.2.2. Exclusion Criteria

Since 2009, the NHIS health examination data has included full lipid profiles (including HDL-C, LDL-C, and TG). Therefore, data from 2002 to 2008, in which only TC levels were collected, was excluded. To ensure consistency in observation duration in the dataset for survival analysis, data from 2011 to 2019 was also excluded.

Individuals diagnosed with stroke, ischemic heart disease, liver disease, sepsis, or cancer during the wash-out period before blood testing were excluded. As the results may have been influenced by previous KM treatment, subjects who used KM treatment during the wash-out period were also excluded.

Among KM users, those with a single KM visit or multiple visits spaced more than 6 weeks apart in the medical treatment history table (20T) for the enrollment period were excluded as temporary users.

#### 2.2.3. Operational Definition

1. Operational definition for KM utilization: KM utilization was defined as having ≥2 sessions of Korean Medicine clinic visits within a 6-week window during the 12-month enrollment period. This threshold was established based on prior NHIS database studies to ensure a balance between treatment adherence and sample representativeness [14]. While acute musculoskeletal conditions often require visits within 2–3 weeks, this study focused on comprehensive health management and herbal medicine interventions, which typically follow clinical cycles of 3 to 6 weeks. Participants meeting these criteria were assigned to the KM user group, while those with no records were assigned to the non-KM user group. Individuals with only a single visit or visits spaced more than 6 weeks apart were excluded as temporary users to minimize misclassification bias.
2. Operational definitions for hyperlipidemia and hypolipidemia: Participants were categorized into three subgroups—hypolipidemia, normal, and hyperlipidemia—based on their lipid profiles from the 2009–2010 national health examination. The operational definitions were adopted and modified from our previous study [15], which utilized a hierarchical algorithm to capture the comprehensive lipid status of the elderly population. While clinical guidelines for hyperlipidemia are well-established, definitions for hypolipidemia in geriatric research often vary across studies. Therefore, we applied the following standardized criteria to ensure a rigorous classification:
  - Primary Criteria (TC-based): Subjects were initially classified by TC levels: hypolipidemia if TC < 150 mg/dL and hyperlipidemia if TC > 240 mg/dL.
  - Secondary Criteria (Specific lipid profiles): For those with normal TC levels of 150 ≤ TC ≤ 240 mg/dL, we further evaluated individual lipid components:
    - Hyperlipidemia was defined if any of the following were met: LDL-C > 130 mg/dL, TG > 200 mg/dL, or HDL-C > 60 mg/dL.
    - Hypolipidemia was defined if the subject did not meet hyperlipidemia criteria but had: LDL-C < 50 mg/dL, TG < 50 mg/dL, or HDL-C < 40 mg/dL.
    - Normal subgroup: Subjects who met none of the above criteria were defined as the normal subgroup.

#### 2.2.4. Outcomes

The primary outcomes included mortality based on all-cause survival, time to disease diagnosis, and morbidity from ischemic heart disease, stroke, liver disease, sepsis, and cancer. Hazard ratios (HR) of all-cause mortality were calculated for both the KM users and control groups, comparing mortality up to 120 months after the baseline of health examination. Disease diagnoses were classified using ICD codes: ischemic heart disease (I21–25), stroke (I60–64), liver disease (K70–77), sepsis (A40–41), and cancer (C00–80), according to the diagnostic codes proposed by the Health Insurance and Assessment Service (HIRA). The baseline was defined as the first visit date for the KM user group and the first examination date for the control group to address and minimize potential immortal bias that may have provided an immortal period to the KM user group if the first examination date had been used.

Secondary outcomes included medical utilization of outpatient visits, total medical expenses, and statin use. Statin use included medications such as atorvastatin, fluvastatin, lovastatin, pravastatin, simvastatin, rosuvastatin, and pitavastatin, or any combination therapy.

### 2.3. Statistical Analysis

All statistical analyses were performed using SAS version 9.4 (SAS Institute Inc., Cary, NC, USA).

#### 2.3.1. Propensity Score Matching

Propensity score matching (PSM) was performed between the KM user and non-user groups using multivariable logistic regression, adjusting for covariates such as age, sex, body mass index (BMI), Charlson Comorbidity Index (CCI), and International Physical Activity Questionnaire (IPAQ). The CCI, which assesses mortality based on disease history, was calculated using Quan’s method for ICD-10 codes, classifying patients into groups ranging from 1 to 8 [16,17,18,19,20].

#### 2.3.2. Variables and Statistical Conditions

For categorical independent variables, binary dependent variables were analyzed using the chi-square test or Fisher’s exact test, whereas continuous dependent variables were assessed using a two-sample independent *t*-test if normality was satisfied. For three or more subgroups, analysis of variance (ANOVA) was employed. If normality was not met, the Wilcoxon rank-sum test was conducted to evaluate the impact of independent variables on dependent variables.

Age stratification included the early elderly group (65–74 years), the middle elderly group (75–84 years), and the late elderly group (85 years and older). Independent variables included serum lipid levels from the health examination table, and the frequency and duration of Korean Medicine utilization from the medical treatment table. Dependent variables consisted of data from the mortality table, including the date of death, cause of death, diagnosis details from the medical treatment table, and key examination results from the health examination table.

The significance level was set at α = 0.05 for two-tailed tests, with a 95% confidence interval (CI). AIC model selection was used to distinguish between a set of possible models describing the relationship between age, sex, and body mass index. The best-fit model, carrying 96% of the cumulative model weight, included all the parameters with no interaction effects.

#### 2.3.3. Descriptive and Frequency Analysis

Descriptive statistical analysis was conducted to determine the frequency, percentile, mean, and standard deviation of the demographic factors, independent variables, and dependent variables. Outliers, defined as values outside 1.5 times the interquartile range (IQR), were excluded.

Comparisons between KM users and controls in terms of demographic characteristics (including age, sex, income from the eligibility table, BMI, dyslipidemia, CCI, and IPAQ) from the medical treatment table and health examination table were made using the chi-square test and Student’s *t*-test.

#### 2.3.4. Survival Analysis and Kaplan–Meier Analysis

HR and 95% CI between groups were calculated using the Cox proportional hazards model. Survival times and cumulative incidence of the diagnosis rates were calculated using Kaplan–Meier analysis.

#### 2.3.5. Multivariable Regression Analysis

To identify independent predictors of all-cause mortality, a multivariable logistic regression analysis was performed. Odds ratios (ORs) and 95% CIs were calculated to assess the association between mortality risk and various factors, including KM utilization, age, sex, BMI, CCI, and physical activity (IPAQ). This analysis complemented the survival analysis by evaluating the overall contribution of each factor to mortality outcomes within the 120-month follow-up period.

### 2.4. Ethics Approval

The database analyzed herein was generated from claims data and, thus, was not subject to consent acquisition. All personal information is masked by the NHIS prior to its public release. This study was conducted in accordance with the Declaration of Helsinki and was approved by the Public Institutional Review Board of the Korean National Institute for Bioethics Policy on 23 August 2024. (Approval number: P01-202408-01-040).

## 3. Results

### 3.1. Subject Characteristics

**Figure 2:**
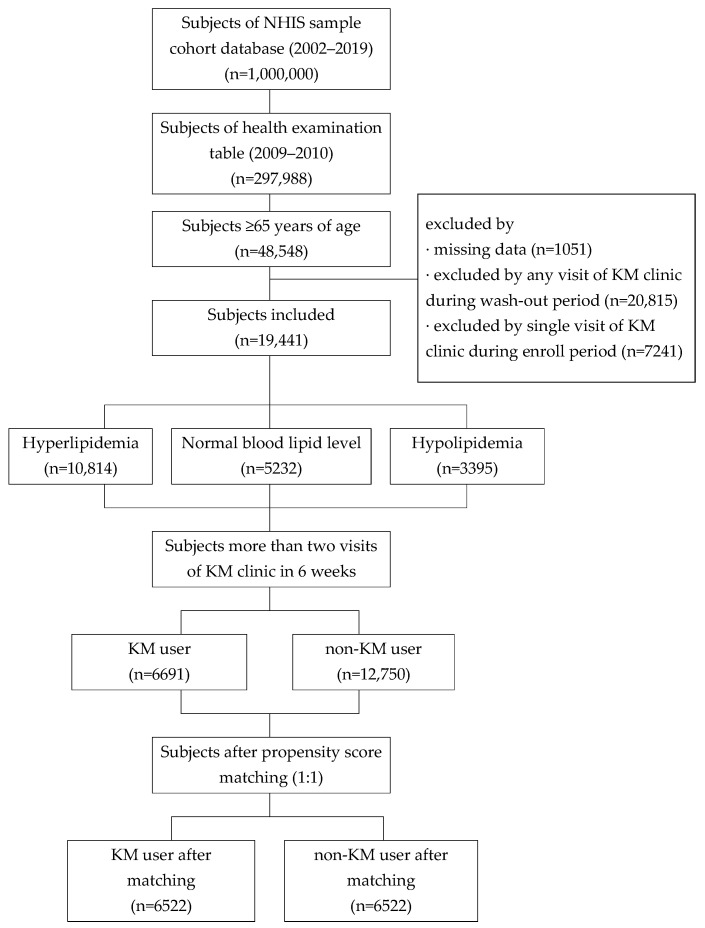
Flow diagram of data selection process.

A total of 48,548 elderly individuals (aged 65 or older) received health examinations. After applying the exclusion criteria, 19,441 patients were eligible for this study. Among these, 6691 patients were eligible for inclusion in the KM user group, and 12,750 patients were eligible for the non-KM user group. After PSM, 13,044 participants were included in this study, with 6522 patients in each group (Table 1, Figure 2).

### 3.2. Mortality and Survival Time

**Figure 3:**
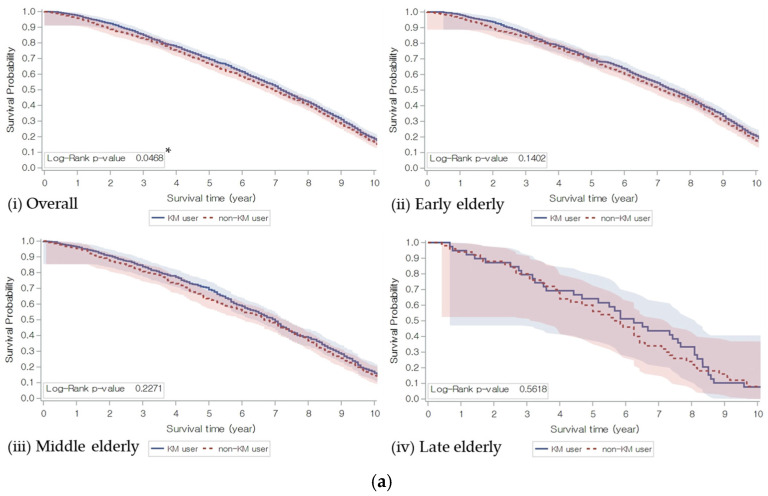

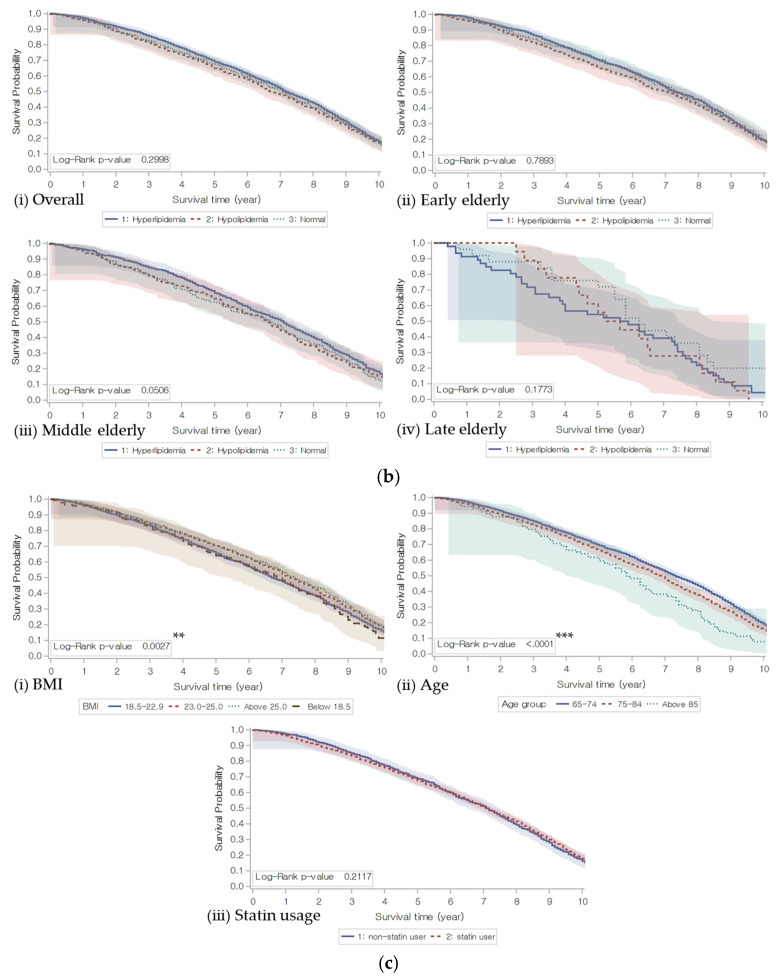
Kaplan–Meier survival estimates of all-cause mortality. (**a**) Group by Korean Medicine utilization; (**b**) Group by dyslipidemia; and (**c**) Group by BMI, age, and statin usage. Estimated all-cause survival probability for the elderly patient by KM treatments, dyslipidemia, and age groups is shown in each graph. *p*-value was calculated from log-rank test (* *p* < 0.05, ** *p* < 0.01, and *** *p* < 0.001). X-axis: survival time (years); and Y-axis: estimated survival probability (%).

Kaplan–Meier curves showed that the KM user group had significantly increased all-cause survival times compared to the control group. (HR 0.93; 95% CI 0.87–1.00, *p* = 0.048). In the subgroup analysis, the hypolipidemia subgroup did not show significant differences in all-cause mortality and survival time compared to the normal and hyperlipidemia subgroups. By the age groups, the mortality of the KM user group was significantly lower in subjects aged 85 and older compared to the control group. Among the BMI groups, the low BMI group showed the highest mortality and a significantly decreased survival time compared to the other BMI groups, but there were no significant differences between KM users and non-users. In the statin use group, KM users on statins showed a statistically significant improvement in all-cause survival time compared to the control group (Table 2) (Figure 3).

**Figure 4:**
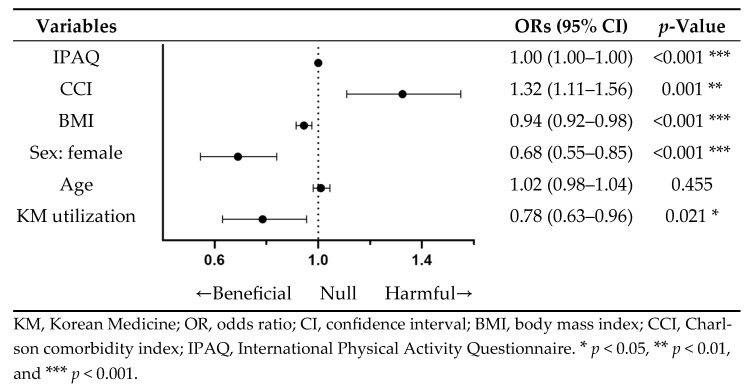
Multivariable logistic regression analysis of all-cause mortality risk associated with various factors.

In multivariable logistic regression analysis, KM utilization was significantly associated with a reduced risk of mortality (OR 0.78; 95% CI 0.63–0.96; *p* = 0.021). This protective effect of KM utilization on mortality was more pronounced than that of BMI (OR 0.94; 95% CI 0.92–0.98; *p* < 0.001). Conversely, a higher CCI was positively associated with increased mortality risk (OR 1.32; 95% CI 1.11–1.56; *p* = 0.001), and female sex showed a lower risk of mortality compared to males (Figure 4).

### 3.3. Morbidity and Time to Disease Diagnosis

The incidence probabilities of all types of diseases, including stroke, coronary artery disease, and liver disease, were higher in the KM user group. The differences were significant for all types of diseases (HR 1.09; 95% CI 1.04–1.14, *p* < 0.001) and stroke (HR 1.10; 95% CI 1.02–1.19, *p* = 0.017), whereas in the other morbidity subgroups, there were no significant differences between the groups.

**Figure 5:**
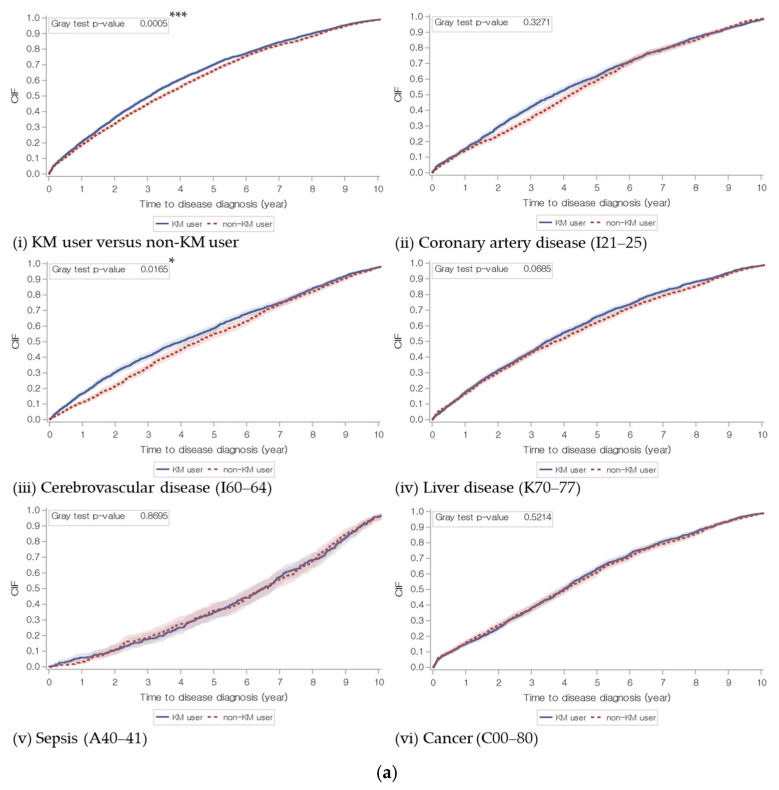

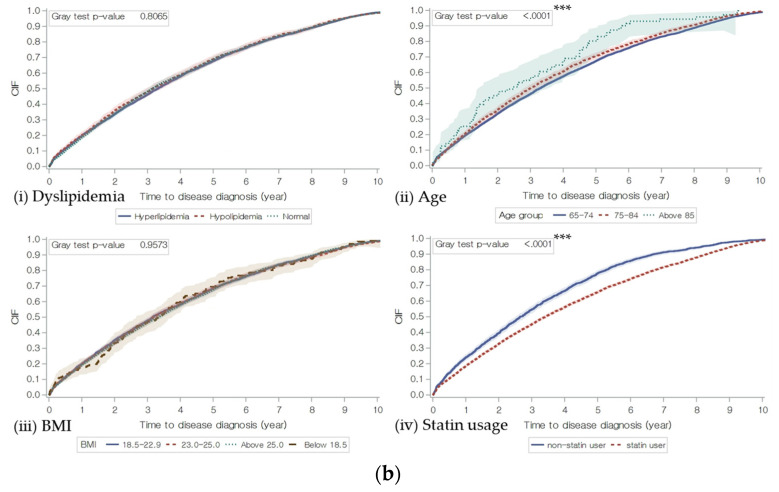
Cumulative incidence risk estimates of disease diagnosis. (**a**) Group by Korean Medicine utilization and type of disease; (**b**) Group by dyslipidemia, age, BMI, and statin usage. Estimated cumulative incidence of disease risk for the elderly patient with and without KM treatments is shown in each graph. *p*-value was calculated from Gray’s test (* *p* < 0.05 and *** *p* < 0.001). X-axis: time to disease diagnosis (years); and Y-axis: estimated cumulative incidence function of disease diagnosis (%).

In the subgroup analysis, the hypolipidemia and hyperlipidemia subgroups showed significantly increased overall disease morbidity and shorter time to disease diagnosis in the KM user group. By the age groups, the morbidity of the KM user group was significantly lower than that of the control group in subjects aged from 65 to 75 years old. Among the BMI groups, the normal BMI group showed higher morbidity in the KM user group than in the control group, and the overweight BMI group had a shorter time to disease diagnosis in the KM user group than in the control group. In the statin use group, both statin users and non-users showed increased morbidity and a reduced time to disease diagnosis with KM use, though the HR was lower in the statin user group (Table 3, Figure 5).

### 3.4. Medical Utilization, Expenses, and Statin Usage

Analysis of healthcare utilization revealed that the KM users had 93 more days of healthcare utilization compared to non-users. The total medical expenses for the KM user group were 5,320,000 KRW higher than for the non-user group, which also demonstrated a statistically significant difference. However, no significant differences in statin usage were observed, with both groups reporting 1.6–1.7 prescriptions.

Regarding medical utilization based on the presence of dyslipidemia, no significant differences were found between groups. Nevertheless, medical expenses were significantly higher in the hypolipidemia subgroup (over 3,000,000 KRW). Statin usage was also highest in this group, with a difference of more than 0.6 prescriptions compared to other lipid status subgroups (Table 4).

## 4. Discussion

### 4.1. General Findings

This study analyzed 13,044 elderly subjects to investigate the impact of KM utilization and lipid status on long-term health outcomes. Our survival analysis and multivariable regression revealed that KM use was significantly associated with reduced all-cause mortality, even after adjusting for covariates such as BMI and comorbidities. While the absolute difference in mean survival time was approximately several months, suggesting a subtle clinical impact on overall life expectancy, the findings indicate that KM utilization is associated with slightly prolonged survival even in the presence of major chronic conditions like stroke, CAD, and liver disease. This suggests that KM may play a potential supportive role in geriatric health management.

In the context of dyslipidemia, the hypolipidemia subgroup exhibited no significant difference in mortality rates and survival times, compared to both the normal lipid and hyperlipidemia subgroups. Mortality rates based on survival time were analyzed using Kaplan–Meier survival curves and the log-rank test. Significant differences were noted in the utilization of KM and the presence of hypolipidemia or hyperlipidemia. Notably, the disparity in mortality rates over time was not proportional, with a notable reduction in the gap observed at the ten-year mark.

### 4.2. Clinical Implications

Regarding dyslipidemia, the hypolipidemia subgroup showed no significant differences in mortality or survival compared to the normal lipid level or hyperlipidemia subgroups. Notably, the KM survival curves revealed that the mortality in the hyperlipidemia subgroup was slightly lower than that for the normal lipid subgroup, which contrasts with traditional expectations of lipid-related cardiovascular risk. This unexpected survival pattern in the hyperlipidemia subgroup suggests that other factors, such as the proactive use of lipid-lowering medications among those diagnosed with hyperlipidemia, may influence survival outcomes. In these cases, the risk profile of a treated hyperlipidemic patient may not be directly comparable to that of a normolipidemic individual not receiving treatment. These results underscore the need for further research into the synergistic interactions between blood lipid levels, pharmacological interventions like statins, and KM utilization.

Furthermore, our analysis of BMI subgroups highlighted that individuals with low to normal BMI faced higher mortality and shorter survival compared to the overweight or obese groups. Since morbidity rates did not differ significantly across BMI categories, this survival disadvantage likely reflects a lack of physiological reserve or physical capacity to cope with illness in underweight older adults. Consequently, close surveillance of BMI and nutritional status is essential for vulnerable elderly populations.

### 4.3. Interpretation of the Associations Between Lipid Status and Clinical Outcomes

Our findings revealed that the hypolipidemia subgroup faced significant disadvantages in health outcomes, including a higher incidence of diseases and increased medical expenses. This is consistent with the concept of reverse epidemiology in the elderly, where low lipid levels often serve as surrogate markers for frailty, malnutrition, or a heavy underlying disease burden. Furthermore, our previous scoping review highlighted that hypolipidemia is frequently associated with severe illness, intensive care unit hospitalization, and immunocompromised states related to altered lipid metabolism in conditions such as liver disease or malignancy [15]. Notably, recent evidence suggests that excessively low LDL-C concentrations may increase bleeding risk, potentially contributing to adverse outcomes in geriatric populations [21].

Additionally, subgroup analyses indicated that statin usage significantly influences health outcomes. While the time-dependent interplay between statin prescriptions and fluctuating lipid levels remains complex, the observed survival benefits may be attributed to the pleiotropic effects of statins, such as their antithrombotic and anti-inflammatory properties [22]. Such mechanisms are particularly relevant in the elderly, for whom chronic inflammation and thrombogenesis are key determinants of long-term prognosis.

Despite the use of PSM, residual confounding factors may persist. Selection bias and unmeasured confounders may influence the estimated effect of KM utilization on geriatric health outcomes. Therefore, the results regarding survival and disease incidence should be interpreted with caution, as we cannot entirely exclude the influence of baseline conditions such as activities of daily living (ADL), compliance with medical advice, behavioral patterns, or socioeconomic status. Nevertheless, KM utilization might act as a mediator for better healthcare accessibility and healthier lifestyle behaviors, especially within the context of the Korean National Health Insurance system, which covers a significant portion of KM services. Unlike controlled trials, real-world evidence (RWE) reflects the comprehensive and mediating effects of multiple intersecting factors on health outcomes. From this perspective, the interaction between KM utilization and patient-specific factors may influence mortality and disease incidence through complex mediating pathways.

### 4.4. Theoretical Implications from Korean Medicine

*Yangseng* (traditional concept of wellbeing) is a core concept in Korean Medicine for promoting healthy aging among the elderly. In the clinical care of the elderly during Korean Medicine utilization, assessment regarding *deficiency and excess*, diet, exercise, and daily life is frequently included [23,24,25]. These differences in healthcare utilization characteristics may have contributed to the observed differences in health outcomes based on the utilization of Korean Medicine. In addition to chronic diseases and multimorbidity, elderly individuals are likely to face mental, physical, and social risk factors such as cognitive impairment, sleep disorders, deterioration of personal hygiene, or physical activity [26,27,28,29]. The comprehensive approach and care using Korean Medicine may serve as a potential mechanism contributing to the improvement of health outcomes among KM users.

### 4.5. Frailty and Geriatric Vulnerability

A defining clinical characteristic of the elderly is frailty, described as a state of increased vulnerability resulting from age-associated declines in reserve and function across multiple physiologic systems [30]. This geriatric vulnerability plays a pivotal role when patients encounter acute stressors or chronic comorbidities. While various diagnostic tools such as the frailty phenotype and index have been introduced [31], detailed patient-reported information or physical function measures are often omitted in claims-based datasets like the NHIS.

Nevertheless, frailty may be the missing dimension that explains the observed “lipid paradox”, negative outcomes of the hypolipidemia subgroup in our study. In geriatric populations, low lipid levels frequently do not represent optimal cardiovascular health but rather reflect an underlying state of frailty, malnutrition, or advanced biological exhaustion. This compels a paradoxical relationship between lipid levels and mortality, where traditional risk factors are overshadowed by geriatric vulnerability.

Therefore, clinical decisions regarding the elderly must shift from a lipid-centric approach to a more comprehensive geriatric assessment that accounts for frailty. While we utilized proxy parameters such as BMI, age, and the CCI to adjust for this vulnerability during data analysis, the lack of a standardized frailty data remains a limitation. Future research integrating validated frailty measures is essential to further elucidate these complex health outcomes.

### 4.6. Limitations and Suggestions

The data from the NHIS used in this study were limited to individuals who underwent regular health examinations, which may introduce selection bias, as individuals who did not undergo health examinations and elderly inpatients in medical institutions were not included in the health examination table. While differences between groups can be adjusted using PSM, inherent biases in the original data cannot be corrected, necessitating caution when interpreting the study results.

Additionally, the use of data from the NHIS rather than medical records may lead to an underestimation or overestimation of risks due to the indirect information constructed for medical insurance services. Health outcome studies utilizing prospective or retrospective medical record data can track patients’ presenting symptoms, signs, adverse reactions, and medical histories, along with laboratory test data collected before and after treatment. However, this study was designed based on available and limited data such as health examination data and diagnostic codes. Therefore, it is essential to supplement the evidence of efficacy and safety in future studies involving retrospective medical record analyses or prospective clinical research.

In this study, the survival duration of the group using Korean Medicine was significantly longer; however, the medical utilization and medical expenses were higher. For instance, even in the non-user group, there may be frequent unobserved utilization of Korean Medicine after 12 months, and transitions between the groups may occur due to subsequent statin usage, even if individuals had hypolipidemia or hyperlipidemia at the time of screening. Nevertheless, this study applied the criterion that the group assignments were fixed at screening and did not update the groups during the follow-up period. This suggests that various complex factors may influence the observation of long-term health outcomes for up to 120 months following the use of Korean Medicine within 12 months post-screening, indicating the need for caution in interpreting the study results.

Potential immortal time bias, arising from the operational definition of a 12-month enrollment window for the KM group, may introduce a positive bias in survival outcomes. While we attempted to mitigate this by setting the first KM visit as the index date, this resulted in a temporal mismatch of baselines between groups. Also, since KM users were required to survive until their second visit within the first 12 months, those who expired shortly after the health examination but before meeting the KM criteria were naturally excluded, even if the subject intended to. Nevertheless, our Kaplan–Meier analysis suggests this bias had a minor impact on the overall results, as the survival curves for the KM and non-KM groups did not show immediate or significant separation during the initial 12-month period. Instead, the curves diverged primarily after the early observation phase, indicating that the long-term survival benefit is more likely associated with sustained healthcare utilization rather than a selection artifact. Nevertheless, the difference in index dates (first KM visit vs. examination date) should be considered when interpreting these findings, and future longitudinal studies should aim to further minimize this temporal misalignment.

Regarding the propensity score matching, we verified the balance between groups primarily through *p*-values across clinically meaningful subgroups rather than reporting standardized mean differences (SMD) for all continuous variables. Given that the distribution of key covariates, such as age and BMI categories, showed near-perfect alignment after matching (*p* > 0.05) and their mean values were nearly identical, the potential for residual imbalance is considered minimal. However, we acknowledge that the absence of explicitly reported SMDs may limit a more granular assessment of matching quality, and this should be considered when interpreting the results.

Another point to note is that, while the intention was to observe the long-term health outcomes associated with the utilization of Korean Medicine, this study faced challenges in accurately collecting data on the use of herbal medicine. The data from the NHIS are primarily composed of information related to health insurance benefits, which results in the significant omission of non-covered services or items such as herbal medicine usage. Consequently, it was not possible to evaluate the effects of herbal medicines on mortality, survival duration, or disease incidence. This limitation should be addressed by using more representative real-world data sources or prospective clinical studies. For instance, utilizing healthcare big data from countries where the use of herbal medicine and herbal formulations is prevalent, such as Taiwan and Japan, could facilitate subsequent research to compensate for these limitations and evaluate traditional East Asian medicine (TEAM), including herbal medicine.

To summarize, the elderly population tends to benefit in terms of mortality rates and all-cause survival time when using Korean Medicine and showed poor outcomes with hypolipidemia, underweight, and statin use. However, the limitations of increasing medical utilization and the higher incidence of disease in the group using Korean Medicine suggest that confounding factors were involved in the health outcomes in this study. Therefore, further prospective studies with pragmatic designs are needed to investigate the effects of Korean Medicine on the elderly.

## 5. Conclusions

In the elderly population, KM utilization is associated with a reduced risk of all-cause mortality and improved survival outcomes, particularly among statin users. While KM users showed a higher cumulative incidence of disease diagnosis, this likely reflects increased diagnostic opportunities resulting from extended survival and proactive medical utilization. Additionally, hypolipidemia, being underweight, and being late-elderly (over 85) were identified as significant risk factors for mortality. The impact of Korean Medicine on health outcomes such as medical utilization, chronic diseases, and mortality is associated with complex clinical factors. Further prospective studies are necessary to investigate the effects of Korean Medicine on the elderly.

## Figures and Tables

**Table 1:** Descriptive characteristics.

	Before Propensity Score Matching				After propensity Score Matching			
Variables	Total	KM User	Non-KM User	*p*-Value	Total	KM User	Non-KM User	*p*-Value
*n*	19,441	6691	12,750		13,044	6522	6522	
Age (year)	71.4 ± 4.8	71.3 ± 4.7	71.5 ± 4.9		71.2 ± 4.6	71.2 ± 4.6	71.1 ± 4.6	
Age group (*n*, %)								
Age: 65–74	15,272 (78.6)	5317 (79.5)	9955 (78.1)	0.003 **	10,486 (80.4)	5233 (80.2)	5253 (80.5)	0.823
Age: 75–84	3929 (20.2)	1313 (19.6)	2616 (20.5)		2450 (18.8)	1236 (19.0)	1214 (18.6)	
Age: above 85	240 (1.2)	61 (0.9)	179 (1.4)		108 (0.8)	53 (0.8)	55 (0.8)	
Sex: female (*n*, %)	8621 (44.3)	3804 (56.9)	4817 (37.8)	<0.001 ***	7517 (57.6)	3758 (57.6)	3759 (57.6)	0.986
Income group (*n*, %)								
Low income (1–3rd decile)	3909 (20.1)	1285 (19.2)	2624 (20.6)	0.012 *	2555 (19.6)	1262 (19.4)	1293 (19.8)	0.384
Middle income (4–7th decile)	5942 (30.6)	2013 (30.1)	3929 (30.8)		3968 (30.4)	1960 (30.1)	2008 (30.8)	
High income (8–10th decile)	9590 (49.3)	3393 (50.7)	6197 (48.6)		6521 (50.0)	3300 (50.6)	3221 (49.4)	
BMI (kg/m^3^)	23.7 ± 3.2	24.1 ± 3.2	23.5 ± 3.2		24.1 ± 3.1	24.1 ± 3.1	24.1 ± 3.1	
BMI group (*n*, %)								
BMI: below 18.5	925 (4.8)	217 (3.2)	708 (5.6)	<0.001 ***	312 (2.4)	156 (2.4)	156 (2.4)	0.417
BMI: 18.5–22.9	7178 (36.9)	2239 (33.5)	4939 (38.7)		4415 (33.9)	2174 (33.3)	2241 (34.4)	
BMI: 23–24.9	4924 (25.3)	1747 (26.1)	3177 (24.9)		3380 (25.9)	1729 (26.5)	1651 (25.3)	
BMI: above 25	6414 (33.0)	2488 (37.2)	3926 (30.8)		4937 (37.9)	2463 (37.8)	2474 (37.9)	
Dyslipidemia group (*n*, %)								
Hypolipidemia	3395 (17.5)	1073 (16.0)	2322 (18.2)	<0.001 ***	2068 (15.9)	1034 (15.9)	1034 (15.9)	0.827
Normal	5232 (26.9)	1791 (26.8)	3441 (27.0)		3458 (26.5)	1744 (26.7)	1714 (26.3)	
Hyperlipidemia	10,814 (55.6)	3827 (57.2)	6987 (54.8)		7518 (57.6)	3744 (57.4)	3774 (57.9)	
CCI	2.9	2.9	2.9		2.9	2.9	2.9	
IPAQ (minutes/week)	443.8	434.2	448.8		434.3	436.2	432.5	

BMI, body mass index; CCI, Charlson comorbidity index; IPAQ, International Physical Activity Questionnaire; KM, Korean Medicine. *p*-value was calculated from chi-square test (* *p* < 0.05, ** *p* < 0.01, and *** *p* < 0.001).

**Table 2:** Comparison of all-cause mortality and hazard ratios.

Variables	Total	KM User	Non-KM User	*p*-Value	HRs ^#^ (95% CI)	*p*-Value ^#^
*n*	13,044	6522	6522			
Mortality (*n*, %)	3370 (25.8)	1681 (25.8)	1689 (25.9)	0.873	0.93 (0.87–1.00)	0.048 *
Lipid group: hypolipidemia	701 (33.9)	350 (33.9)	351 (34.0)	0.963	0.93 (0.81–1.06)	0.277
Lipid group: normal	871 (25.2)	427 (24.5)	444 (25.9)	0.336	0.88 (0.76–1.03)	0.116
Lipid group: hyperlipidemia	1798 (23.9)	904 (24.2)	894 (23.7)	0.642	0.95 (0.87–1.05)	0.338
Age group: 65–74	2065 (19.7)	1026 (19.6)	1039 (19.8)	0.824	0.93 (0.86–1.02)	0.143
Age group: 75–84	1216 (49.6)	616 (49.8)	600 (49.4)	0.837	0.93 (0.83–1.04)	0.230
Age group: above 85	89 (82.4)	39 (73.6)	50 (90.9)	0.018 *	0.88 (0.58–1.35)	0.564
BMI group: below 18.5	139 (44.6)	67 (43.0)	72 (46.2)	0.569	0.76 (0.54–1.06)	0.115
BMI group: 18.5–22.9	1404 (31.8)	686 (31.6)	718 (32.0)	0.730	0.93 (0.84–1.03)	0.197
BMI group: 23–24.9	798 (23.6)	410 (23.7)	388 (23.5)	0.885	0.98 (0.85–1.12)	0.735
BMI group: above 25	1029 (20.8)	518 (21.0)	511 (20.7)	0.745	0.93 (0.83–1.05)	0.270
Statin group: user	855 (36.8)	473 (37.4)	382 (36.1)	0.248	0.91 (0.84–0.99)	0.022 *
Statin group: non-user	2515 (23.5)	1208 (23.0)	1307 (23.9)	0.511	0.99 (0.87–1.14)	0.947

KM, Korean Medicine; HR, hazard ratio; CI, confidence interval; BMI, body mass index. *p*-value was calculated from chi-square test (* *p* < 0.05). ^#^ HRs, 95% CI, and *p*-value were calculated from Cox regression analysis and log-rank test.

**Table 3:** Comparison of disease incidence and cause-specific hazard ratios.

Variables	Total	KM User	Non-KM User	*p*-Value	HRs ^#^ (95% CI)	*p*-Value ^#^
*n*	13,044	6522	6522			
Disease diagnosis (*n*, %)	8241 (63.2)	4218 (64.7)	4023 (61.7)	<0.001 ***	1.09 (1.04–1.14)	<0.001 ***
Disease: CAD	2933 (22.5)	1542 (23.6)	1391 (21.3)	0.002 **	1.04 (0.96–1.11)	0.327
Disease: CVA	2606 (20.0)	1415 (21.7)	1191 (18.3)	<0.001 ***	1.10 (1.02–1.19)	0.017 *
Disease: liver disease	3753 (28.8)	1826 (28.0)	1927 (29.6)	0.051	1.06 (0.99–1.14)	0.069
Disease: sepsis	707 (5.4)	370 (5.7)	337 (5.2)	0.202	0.99 (0.85–1.15)	0.870
Disease: cancer	2617 (20.1)	1374 (21.1)	1243 (19.1)	0.004 **	1.02 (0.95–1.11)	0.521
Lipid group: hypolipidemia	2198 (63.6)	1147 (65.8)	1051 (61.3)	0.007 **	1.09 (1.00–1.19)	0.041 *
Lipid group: normal	1279 (61.9)	654 (63.3)	625 (60.4)	0.189	1.03 (0.93–1.15)	0.583
Lipid group: hyperlipidemia	4764 (63.4)	2417 (64.6)	2347 (62.2)	0.033 *	1.09 (1.03–1.15)	0.003 **
Age group: 65–74	6514 (62.1)	3349 (64.0)	3165 (60.3)	<0.001 ***	1.09 (1.04–1.14)	<0.001 ***
Age group: 75–84	1656 (67.6)	836 (67,6)	820 (67.6)	0.961	1.06 (0.96–1.18)	0.213
Age group: above 85	71 (65.7)	33 (69.1)	38 (62.3)	0.455	1.15 (0.71–1.85)	0.581
BMI group: below 18.5	207 (66.4)	106 (68.0)	101 (64.7)	0.549	0.93 (0.70–1.22)	0.607
BMI group: 18.5–22.9	2787 (63.1)	1443 (66.4)	1344 (60.0)	<0.001 ***	1.08 (1.00–1.16)	0.049 *
BMI group: 23–24.9	2143 (63.4)	1099 (63.6)	1044 (63.2)	0.843	1.18 (1.09–1.28)	<0.001 ***
BMI group: above 25	3104 (62.9)	1570 (63.7)	1534 (62.0)	0.206	1.03 (0.96–1.11)	0.367
Statin group: user	1655 (71.2)	919 (72.7)	736 (69.5)	0.095	1.16 (1.05–1.28)	0.003 **
Statin group: non-user	6586 (61.4)	3299 (62.8)	3287 (60.2)	0.006 **	1.05 (1.01–1.11)	0.024 *

KM, Korean Medicine; HR, hazard ratio; CI, confidence interval; CAD, coronary artery disease; CVA, cerebrovascular disease; BMI, body mass index. *p*-value was calculated from chi-square test (* *p* < 0.05, ** *p* < 0.01, and *** *p* < 0.001). ^#^ HRs, 95% CI, and *p*-value were calculated from cause-specific Cox regression analysis and Gray’s test.

**Table 4:** Medical utilization and expenses in the elderly subjects. (**a**) Comparison between KM users and non-KM users. (**b**) Comparison between dyslipidemia subgroups.

**(a)**				
**Variables**	**KM User** **(*n* = 6522)**	**Non-KM User** **(*n* = 6522)**	**MD** **(95% CI)**	***p*** **-Value**
	**Mean (95% CI)**			
Medical utilization (days)	376.2 (369.4–383.0)	282.7 (276.9–288.5)	93.5 (84.6–102.5)	<0.001 ***
Medical expense (10 k KRW)	2518.3 (2444.6–2591.5)	1985.9 (1919.6–2052.1)	532.2 (433.3–631.0)	<0.001 ***
Statin usage (counts)	1.65 (1.46–1.83)	1.72 (1.46–1.98)	0.08 (−0.25–0.40)	0.643
**(b)**				
**Variables**	**Hypolipidemia** **(*n* = 2068)**	**Normal Lipid Level** **(*n* = 3458)**	**HYPERLIPIDEMIA (*n* = 7518)**	***p*** **-Value**
	**Mean (95% CI)**			
Medical utilization (days)	331.8 (320.1–343.4)	322.8 (314.6–331.0)	331.9 (325.7–338.0)	0.229
Medical expense (10 k KRW)	2567.0 (2420.8–2713.1)	2169.7 (2077.7–2261.7)	2203.1 (2139.9–2266.4)	<0.001 ***
Statin usage (counts)	2.35 (1.85–2.84)	1.23 (0.92–1.54)	1.71 (1.52–1.91)	<0.001 ***

(**a**) KM, Korean Medicine; MD, mean difference; CI, confidence interval. *p*-value was calculated from independent *t*-test (*** *p* < 0.001). (**b**) CI, confidence interval. *p*-value was calculated from one-way ANOVA test (*** *p* < 0.001).

## Data Availability

The data that support the findings of this study are available from the corresponding author upon reasonable request.

## Abbreviations

The following abbreviations are used in this manuscript:

ADL	Activities of daily living
AHA/ACC	American Heart Association and the American College of Cardiology
ANOVA	Analysis of variance
BMI	Body mass index
CCI	Charlson comorbidity index
CI	Confidence interval
HDL	High-density lipoprotein
HDL-C	High-density lipoprotein cholesterol
HIRA	Health Insurance and Assessment Service
HR	Hazard ratios
IPAQ	International Physical Activity
IQR	Interquartile range
KM	Korean Medicine
LDL	Low-density lipoprotein
LDL-C	Low-density lipoprotein cholesterol
NHANES	National Health and Nutrition Examination Survey
NHIS	National Health Insurance Service
NHIS-NSC	National Health Insurance Services Sample Cohort
OR	Odds ratio
PSM	Propensity score matching
RWE	Real-world evidence
SMD	Standardized mean difference
TC	Total cholesterol
TEAM	Traditional East Asian medicine
TG	Triglycerides
